# Botulinum Toxin Type A as a Therapeutic Agent in Epilepsy: Attenuation of Neuronal Ferroptosis and Cognitive Dysfunction

**DOI:** 10.1002/brb3.70930

**Published:** 2025-11-12

**Authors:** Shuang Li, Zhi Huang, Yunqing Ma, Yake Zheng, Maiqi Du, Yajun Lian

**Affiliations:** ^1^ Department of Neurology The First Affiliated Hospital, Zhengzhou University Zhengzhou China; ^2^ Department of Stroke ICU The First Affiliated Hospital, Zhengzhou University Zhengzhou China

**Keywords:** BoNT/A, cognitive function, ferroptosis, neuroprotective

## Abstract

**Purpose:**

Epilepsy is one of the most common neurological disorders with no effective drugs to prevent seizures or their progression. Iron modulation is a potential advanced treatment for seizures. We aim to investigate whether botulinum toxin type A (BoNT/A) can attenuate epilepsy‐induced neuronal death and maintain cognitive function by inhibiting ferroptosis.

**Method:**

We established an epileptic rat model and intervened with BoNT/A to assess its influence on cognitive functions and the pathological damage of hippocampal tissues. Rat hippocampal neuronal cells were treated with magnesium‐free induction solution to establish an epileptic cell model and intervened using BoNT/A. Changes in ferrous ions (Fe^2+^), malondialdehyde (MDA), and glutathione (GSH) were detected in hippocampal tissues and cells. Western blot (WB) and RT‐qPCR were used to detect the protein expression of the iron death markers, including GPX4, ACSL4, and SLC7A11.

**Finding:**

We found that BoNT/A attenuated epileptiform behavior and cognitive deficits and ameliorated hippocampal tissue damage in rats under lithium chloride‐pilocarpine‐induced epilepsy. In vitro BoNT/A treatment exerted potent neuroprotective effects on hippocampal neuronal cells treated by magnesium‐free induction solution. These protective effects were related to the regulation of ferroptosis mediated by the GPX4/ACSL4/SLC7A11 proteins.

**Conclusion:**

These results suggest that BoNT/A is effective in preventing epileptic neuronal iron death and attenuates cognitive dysfunction through the ferroptosis pathway.

## Graphical abstract

By establishing an epileptic rat model and in vitro epileptic cell model intervening with BoNT/A, we found that BoNT/A is effective in preventing epileptic neuronal iron death and attenuates cognitive dysfunction through the ferroptosis pathway.

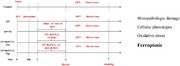

## 1 Introduction

Epilepsy is a complex neurological disorder with diverse etiologies that affects approximately 50 million people worldwide each year (Razaz et al. 2017) and can be triggered by a variety of factors and brain‐associated diseases (Reddy et al. 2017; Reddy 2011; Samba Reddy 2017). Although several antiepileptic drugs have been marketed, they are not indicated for all patients, and many have side effects, indicating no effective drugs for epilepsy (Pauletti et al. 2019). Therefore, it is vital to investigate new therapeutic targets and drugs to delay or prevent seizures, inhibit their progression, and mitigate associated complications.

A new idea is to use botulinum neurotoxins (BoNTs) to promote the repair of nerve damage, especially botulinum toxin type A (BoNT/A), which can inhibit neurotransmitter release by cleaving synaptosome‐associated protein 25 (SNAP25) (Martin et al. 2024). It has been shown that the cleavage of SNAP25 by BoNT/A lasts up to 160 days, a property that has led to its significant impact in clinical applications, such as the treatment of muscle spasms and other disorders (Antonucci et al. 2008; Barr et al. 2005). In our previous study, we investigated the effects of BoNT/A on hippocampal neuronal damage following status epilepticus (SE) induced by hairy fruit rutinosus (Huang et al. 2019). By administering BoNT/A, the expression of caspase‐3 and Bax could be effectively suppressed while attenuating the decrease in Bcl‐2 levels, thereby significantly reducing SE‐induced hippocampal neuronal mortality, indicating the potential application of BoNT/A in the antiepileptic field. These results demonstrate that BoNT/A can inhibit cell death, probably including ferroptosis, to protect the hippocampus from damage. However, the specific mechanism of action of BoNT/A in resisting hippocampal neuronal damage after epilepsy is still unclear.

Ferroptosis is a recently discovered cell death process involving iron overload and lipid peroxidation. Studies have shown the underlying mechanisms between ferroptosis and its associated various brain diseases (Yan et al. 2021), including neurological disorders, stroke, cerebral hemorrhage, and epilepsy (Li et al. 2024 Wang et al. 2024 and Bao et al. 2020), indicating that BoNT/A protects the hippocampus from injury, probably by modulating the ferroptosis pathway. Ferroptosis‐specific inhibitors are effective in restoring neuronal loss in SE (Chen et al. 2022). Through the use of these inhibitors, lipid peroxidation levels in brain tissue can be significantly reduced, thereby attenuating the resulting cellular damage (Ye et al. 2020). In addition, such drugs may also provide protection to damaged neurons by modulating the inflammatory response and improving blood–brain barrier function (Mao et al. 2019). Therefore, exploring the mechanism of neuronal ferroptosis in the brain of epilepsy patients will provide an important reference for understanding their pathobiology.

In this study, we performed in vivo and in vitro experiments to explore how BoNT/A protects hippocampal neurons from damage and identify the downstream targets of BoNT/A. By detecting the expression levels of key factors, we partly identified the underlying mechanism of BoNT/A in neuron protection. The results of this study will not only help to reveal the underlying mechanisms of disease development but also lay the foundation for developing new therapeutic strategies in the future.

## 2 Materials and Methods

### 2.1 Construction of Epilepsy Animal Model

The 6‐8‐week‐old SD male rats (weight 200 ± 20 g) were purchased from Spearfish (Beijing) Biotechnology Co. During the course of this study, all rats were reared ad libitum under standard laboratory conditions. The experimental protocol has been approved by the Ethics Committee of the First Affiliated Hospital of Zhengzhou University with approval number 2023‐KY‐1341.

All SD rats first received lithium chloride (127 mg/kg), followed by pilocarpine (50 mg/kg) administered via intraperitoneal injection 18 h later. Seizure induction was assessed using the Racine score (> 3 as SE) (Luttjohann et al. 2009) and then sustained by injection of diazepam (8 mg/kg) after 60 min. Racine is graded on five levels: Grade I is characterized by slight tremors or jitter, usually limited to specific parts of the movement, and does not have a significant effect on overall behavior; Stage II shows significant forelimb or hind limb shaking, and the animal maintains its posture despite abnormal movement; Grade III is characterized by systemic tremor, when the animal loses the ability to stand but still has partial postural control; Stage IV is characterized by severe generalized tonic–clonic seizures in which the animal completely loses postural control and often rolls on the ground; Grade V is a continuous tonic–clonic seizure, accompanied by a complete loss of consciousness and postural control, with severe symptoms such as convulsions and tremors, and with widespread movement disorders.

All rats were carefully monitored to prevent asphyxia. Typically, spontaneous epilepsy occurs within 14 days of pilocarpine injection.

### 2.2 Animal Grouping Interventions

The experimental rats were randomly divided into five groups with a total of 30 rats. The specific groups were as follows: control group, epilepsy group (EP group), epileptic rats with normal saline group (EP+NS group, epileptic rats with normal saline injection), epileptic rats with hippocampal injection of BoNT/A group (EP+BoNT/A‐Hip group), and epileptic rats with nasal infusion of BoNT/A group (EP+BoNT/A‐Nas group, epileptic rats with nasal infusion of BoNT/A). The 100 U BoNT/A (100 U/bottle, Lanzhou Institute of Biological Products Co. Ltd.) was dissolved in 320 µL of saline (0.9%). Rats were anesthetized using sodium pentobarbital (50 mg/kg, intraperitoneal injection) and kept in a supine position to ensure that the head was parallel to the horizontal plane. In the EP+BoNT/A‐Nas group, each rat was given 80 µL of BoNT/A (25 U) daily by alternating 8 µL drops of the drug into the right and left nostrils, which were replaced every 6 min according to a previous study (Holzmann et al. 2012). During the administration period, the mouth and contralateral nostrils were closed to promote natural inhalation of the drug. This nasal dosing regimen lasted for 3 days, so that each rat received a total of 75 U of BoNT/A. In addition, in the EP+BoNT/A‐Hip group, a single injection of 8 µL of BoNT/A (25 U) was administered via hippocampal stereotaxic localization technique (CA1 region: −3.6 mm in AP coordinates; ±2.5 mm in ML coordinates; −2.0 mm in DV coordinates). In the saline group, an equal amount of saline was given transnasally.

### 2.3 Behavioral Evaluation

Morris water maze: The water maze experiment was performed 72 h after nasal administration. A black circular pool with a diameter of 250 cm and water temperature at 22°C–26°C. During the first 5 days, four trials per day were conducted to train the rats to swim from different starting positions to the underwater platform. The time it took each rat to find the platform was recorded. The platform was removed on Day 6; the number of times the original platform location was traversed within 60 s and the amount of time the rat spent in the target quadrant were recorded.

Open field test: animals were gently placed in the center of the open field box (50 cm × 50 cm). The rats were allowed to move freely in the open field for 15 min. A video surveillance system was used to record the animal's trajectory, residence time, and area of activity.

### 2.4 Histopathological Staining

The rats were euthanized using the cervical dislocation method. For HE staining, the brain hippocampal tissues were removed and processed for paraffin embedding. After being baked, dewaxed, and hydrated, the tissues were stained with hematoxylin staining solution for 3–5 min and stained with eosin for 3–5 min. The sections were dehydrated, sealed, and observed under an inverted microscope.

For Nissl staining, the brains were subsequently removed and processed for paraffin embedding. Paraffin‐embedded brains were cut into coronal sections of 5 µm thickness, and six sections were selected at 50 µm intervals for Nissl staining using toluidine blue.

For Golgi staining, the fixed brain tissues were intercepted at 2 mm thickness and stained with equal solutions A and B at room temperature, avoiding light for 14 days. Then we dehydrated the brain tissue in solution C at 4°C, then used a Golgi‐specific slide to patch the brain tissue and waited for it to dry naturally. The slices were placed in color‐developing solution for 10 min and sealed with glycerol gelatin.

### 2.5 Transmission Electron Microscopic Observation of Ultrastructural Changes in the Hippocampus

Hippocampal tissues were fixed in 2.5% glutaraldehyde for 2 h, treated with 1% Flemming fixative solution for 2 h, and dehydrated at 4°C. Then the tissues were embedded using epoxy resin, and after incubation at 37°C overnight, 45°C for 12 h, and 60°C for 48 h, respectively. Sections were made through an ultrathin sectioning machine with a thickness of 70 nm. Finally, double staining was performed using 3% uranyl acetate‐lead citrate and observed under a transmission electron microscope (JEM‐1230 (80 KV), JEOL, Japan).

### 2.6 Isolation and Culture of Rat Primary Hippocampal Neuronal Cells

Within 24 h of neonation, the bilateral hippocampi of 10 SD rats were separated by a blunt method and cut into small pieces, which were digested by 0.25% (g/mL) trypsin solution. Then a few drops of DMEM/F12 (1:1) medium were added to terminate the digestion process. After centrifugation at 800 r/min for 5 min at 4°C, DMEM/F12 medium was added again for centrifugation. Neurons with a slow apposition rate were collected after 1 h of standing in a CO_2_ incubator, and inoculated on coverslips in 24‐well culture plates pre‐coated with PLL at a density of 1 × 10^5^ cells/mL. The cell growth was observed under an inverted microscope, and by Day 10, the cells could be used for further experimental studies. Cellular NSE (neuronal specific enolase) expression was observed by immunofluorescence.

### 2.7 Cell Grouping Interventions

The hippocampal neuron cells of the third to fifth generation were taken for subsequent experiments. In summary, the cells were divided into the following four groups: (1) control group (control); (2) magnesium‐free inducer group (model); (3) magnesium‐free inducer + 0.05 U/mL BoNT/A group (model + BoNT/A‐0.05 U); and (4) magnesium‐free inducer + 0.1 U/mL BoNT/A group (model + BoNT/A‐0.1 U), which were cultured in corresponding medium.

### 2.8 CCK‐8 Assay for Cell Proliferation Activity

Cell viability was determined using Cell Counting Kit‐8 (CA1210, Solarbio, China). Light absorption values were detected at 450 nm using a microplate reader (Bio Tek, USA).

### 2.9 Fe^2+^, GSH Level, and MDA Level Detection

Hippocampal tissue homogenates were prepared, and the Fe^2+^ assay kit (E‐BC‐K773‐M, Elabscience, China), GSH assay kit (BC1175, Solarbio, China) and MDA content assay kit (BC0025, Solarbio, China), were used to detect Fe^2+^, GSH, and MDA content, respectively, in strict accordance with the respective kit's instructions.

### 2.10 Real‐Time Quantitative PCR

Total RNA from cell cultures was extracted using TRIzol reagent (R1100, Solarbio, China) according to the manufacturer's protocol. Then, RNA was reverse‐transcribed to cDNA using the One Step Super RT‐PCR Mix Kit (T2200, Solarbio, China). Double‐stranded DNA dye SYBR Green (#RR037A; Takara Biotechnology, Dalian, China) was used on an ABI7500 platform. All samples were analyzed in triplicate, and gene expression was normalized to GAPDH. Primer sequences are shown in Table 1 below.

**TABLE 1:** Primer sequences.

Primer	Primer sequences (5′–3′)
GPX4‐F	ATCGATGGGCACATGGTTTG
GPX4‐R	TTCGTAAACCACACTCGGCG
ACSL4‐F	TGCTGCCTGTCCACTTGTTA
ACSL4‐R	AGTCGAAGTGCGTGACAGAG
SLC7A11‐F	GAGTCTGGGTGGAACTGCTG
SLC7A11‐R	CCAGCTGACACTCGTGCTAT
GADPH‐F	GCTGAGAATGGGAAGCTGGT
GADPH‐R	TCTCCATGGTGGTGAAGACG

### 2.11 Western Blot

RIPA lysate containing protease inhibitor was added to fully grind to extract total proteins, and after standing for 1 h, the supernatant was centrifuged at 12000 r/min for 15 min to take the supernatant, and the protein concentration was determined and normalized with a BCA kit to prepare a protein uploading solution with a content of 3 g/L. Gel preparation, sample loading, electrophoresis, membrane transfer, closure, and incubation of primary antibody (ACLS4 [22401‐1‐AP, Proteintech], GPX4 [30388‐1‐AP, Proteintech], SLC7A11 [26864‐1‐AP, Proteintech], and cleaved SNAP25 [GTX39119, GeneTex]) overnight at 4°C were done. The membranes were washed with TBST for 5 min for six times on the second day, added with the secondary antibody, and incubated in the oven at 37°C for 1 h. The membrane was washed by TBST for 5 min for six times. We prepared the development solution, developed the image with a chemiluminescent instrument, and analyzed the protein bands by ImageJ.

### 2.12 Statistical Analysis

SPSS 26.0 statistical software was applied, and the measurements were expressed as mean ± SD. Independent sample Student's *t*‐test was used for comparison between two groups, and one‐way analysis of variance (ANOVA) was used for comparison between multiple groups, followed by Tukey's post hoc test. *p* < 0.05 was taken as the difference was statistically significant.

## 3 Results

### 3.1 BoNT/A Intervention Improves Cognitive Behavior and Suppresses Seizures in Epileptic Rats

**FIGURE 1:**
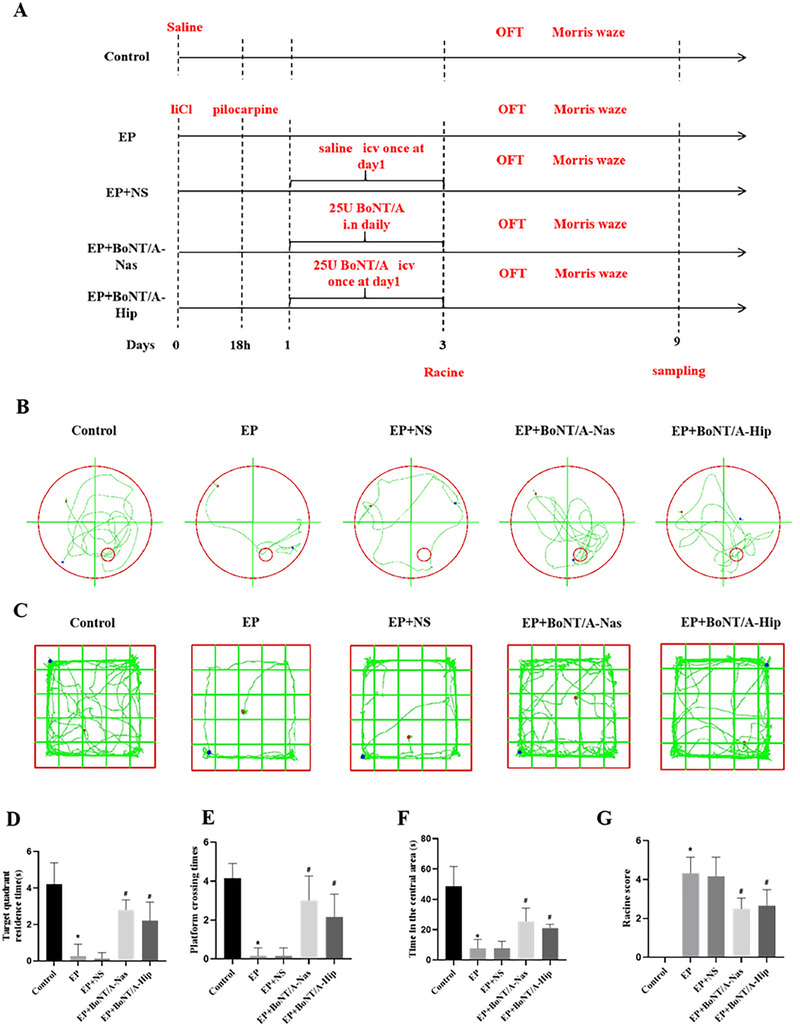
BoNT/A intervention improves cognitive behavior and suppresses seizures in epileptic rats. (A) Schematic diagram of animal experiment procedure. (B) Trajectory diagram of water maze experiment. (C) Trajectory diagrams of the open field experiment in each group of rats. (D) Comparison results of the residence time in the target quadrant for each group of rats. (E) Comparative results of the number of times rats crossed the platform in each group. (F) Comparison results of the residence time in the middle region for each group of rats. (G) Results of Racine scores of rats in each group. *p < 0.05 compared with control group; ^#^ *p *< 0.05 compared with EP group.

To explore how BoNT/A treatment affects the behaviors of epileptic rats, we constructed an epilepsy animal model with BoNT/A intervention and assessed their behavior changes (Figure 1A). The spatial memory behavior of rats in each group was assessed by the Morris water maze experiment, and the main assessment indexes were the residence time in the target quadrant and the number of times the rats crossed the platform (Figure 1B,D,E). Compared with the control group, the residence time in the target quadrant and the number of times crossing the platform were significantly lower in the EP group (*p *< 0.05). Between the EP and EP+NS groups, the residence time in the target quadrant and the number of times crossing the platform did not show significant differences. In contrast, the dwell time and the number of times across the platform in the target quadrant were significantly higher (*p *< 0.05) in the EP+BoNT/A‐Nas group compared to the EP group, and similar results were detected (*p *< 0.05) in the EP+BoNT/A‐Hip group compared to the EP group (Figure 1C,D). Spontaneous activity of rats in each group was assessed by the absent‐field test (Figure 1C,F). The time spent in the intermediate area was significantly lower in rats of the EP group compared with the control group (*p *< 0.05), but did not show a significant difference between the EP and EP+NS groups. In contrast, the time spent was significantly higher (*p *< 0.05) in the EP+BoNT/A‐Nas group compared with the EP group, and higher time was observed (*p *< 0.05) in the EP+BoNT/A‐Hip group compared with the EP group. While no significant difference was observed between the EP+BoNT/A‐Nas and EP+BoNT/A‐Hip groups. Seizures were assessed by Racine score in rats of each group (Figure 1G), showing a significantly higher score in the EP group compared to the control group (*p *< 0.05). Racine scores did not show significant differences between the EP and EP+NS groups. In contrast, Racine scores were significantly lower (*p *< 0.05) in the EP+BoNT/A‐Nas group compared to the EP group and in the EP+BoNT/A‐Hip group compared to the EP group. However, there was no significant difference in Racine scores of rats between the EP+BoNT/A‐Nas and EP+BoNT/A‐Hip groups. In summary, these results demonstrated that BoNT/A treatment significantly changed the behaviors of epileptic rats and showed protective effects.

### 3.2 BoNT/A Intervention Ameliorates Histopathologic Damage in the Hippocampus of Epileptic Rats

**FIGURE 2:**
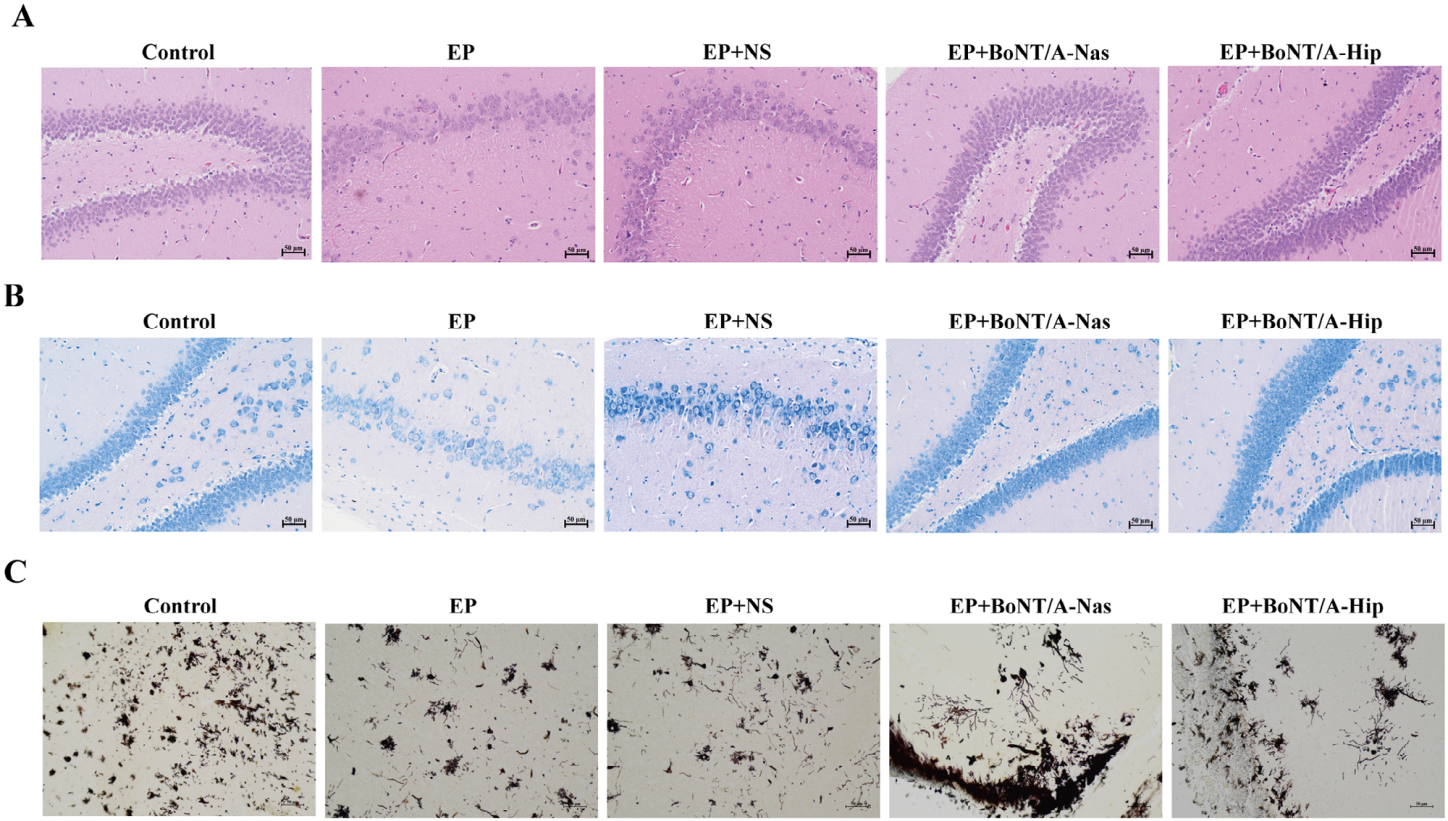
BoNT/A intervention improves hippocampal histopathological damage and hippocampal protrusion plasticity in epileptic rats. (A) Results of HE staining experiments of hippocampal tissues in each group of rats; scale bar is 50 µm (*n* = 3). (B) Results of Nissl staining experiments of hippocampal tissues in each group of rats; scale bar is 50 µm (*n* = 3). (C) Results of Golgi staining experiments of hippocampal tissues in each group of rats; scale bar is 50 µm (*n* = 3).

The cytopathological changes in the hippocampal tissue of rats in each group were observed by HE staining. In the blank control group, the cells in the hippocampal tissue were normal in morphology and neatly arranged, with abundant cytoplasm and large, round nuclei, while in the EP and EP+NS groups, the number of cells in the hippocampal tissue was significantly reduced, the arrangement was loose and disordered, the cytoplasm was darkly stained, and the nuclei appeared to be lysed and fragmented, with an empty vesicle‐like appearance (Figure 2A). The cellular situation in the EP+BoNT/A‐Nas group and the EP+BoNT/A‐Hip group was improved compared with the EP group in cell number and cellular morphology. The EP+BoNT/A‐Hip group showed an improvement in the cellular condition compared with the EP group in cell number and cell morphology. Morphological changes of rat hippocampal neurons in each group were observed by Nissl staining (Figure 2B). The neurons in the control group were neatly arranged and had normal morphology, and Nissl vesicles were clearly visible. In contrast, the neurons in the EP and EP+NS groups were clearly visible, and the neurons in the EP+BoNT/A‐Nas and EP+BoNT/A‐Hip groups were slightly disorganized. Although the cellular morphology was basically kept neat, the staining of the Nissl vesicles was relatively thin, and the cellular gaps were enlarged, but on the whole, most of the neurons still maintained a certain degree of activity and function. In conclusion, BoNT/A treatment can protect the hippocampal tissue from EP‐induced damages and keep the normal morphology of hippocampal cells.

### 3.3 BoNT/A Intervention Improves Hippocampal Synaptic Plasticity in Epileptic Rats

Dendritic spines in the hippocampal region of rats in each group were observed by Golgi staining. First, the total dendritic spine density and the density of mature dendritic spines were significantly reduced in the EP and EP+NS groups compared with the blank group. Further, comparing the EP group with the EP+BoNT/A‐Nas group and the EP+BoNT/A‐Hip group, an increase in total dendritic spine density and mature‐type dendritic spine density was observed in the latter two (Figure 2C), suggesting that neurons in these experimental groups may have undergone a certain degree of recovery or remodeling process after the imposition of BoNT/A treatment, thus promoting the development of dendritic spines.

### 3.4 BoNT/A Intervention Modification Reduces Ferroptosis and Oxidative Stress in Epileptic Rats

**FIGURE 3:**
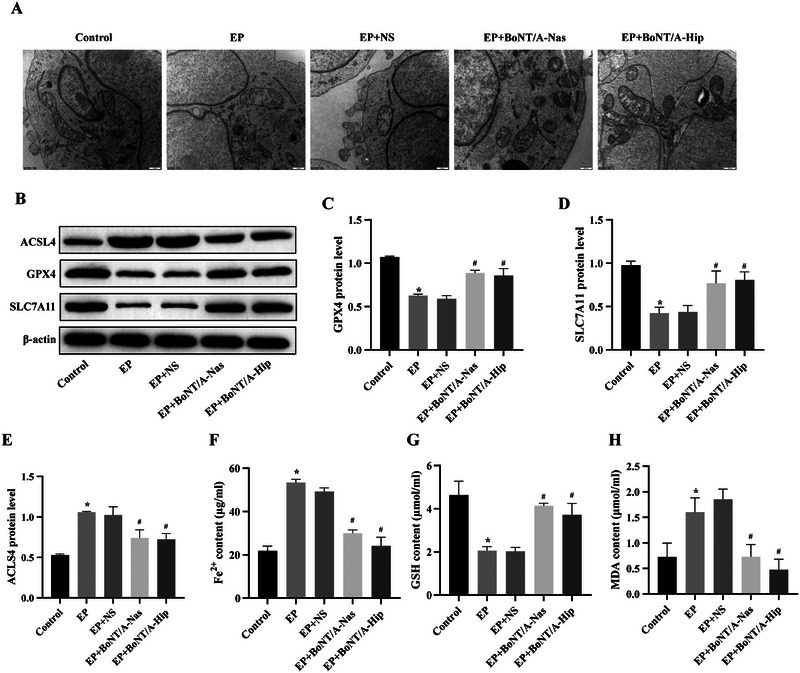
BoNT/A intervention modification reduces iron death and oxidative stress in epileptic rats. (A) Transmission electron microscopy observations of hippocampal tissues of rats in each group with a scale bar of 500 nm. (B) Representative WB assay strip plots. (C–E) Bar graphs of protein expression of GPX4, ACSL4, and SLC7A11. (F) Histogram of Fe^2+^ content results. (G) Histogram of GSH content results. (H) Histogram of MDA content results. Compared with the control group, *p < 0.05; compared with the EP group, ^#^ *p *< 0.05.

The cleaved SNAP25 protein was highly expressed in the BoNT/A‐intervened groups and not in other groups (Figure S1). Changes in the morphology of cellular mitochondria in each group were observed by transmission electron microscopy (Figure 3A). The mitochondrial membrane structure of the control group remained intact and showed the characteristics of healthy cells, with mitochondria showing a typical oval shape and sufficient number. However, in the EP and EP+NS groups, blurring of the nuclear membrane structure and reduction in the number of organelles were found, along with swelling and decrease in the number of mitochondria, accompanied by vacuole‐like changes. Further analysis showed that in the EP+BoNT/A‐Nas and EP+BoNT/A‐Hip groups, a significant reduction in cell swelling was observed, the membrane structure remained intact, and the overall cellular morphology tended to be normalized.

The GPX4, ACSL4, and SLC7A11 protein expression in the hippocampal tissues of rats in each group was detected by WB (Figure 3B–E). Compared with the control group, the protein expression levels of GPX4 and SLC7A11 in the EP group were significantly lower, while the protein expression level of ACSL4 was significantly higher (*p *< 0.05). Between the EP and EP+NS groups, GPX4, ACSL4, and SLC7A11 protein expression levels did not show significant differences. In contrast, the protein expression levels of GPX4 and SLC7A11 in the EP+BoNT/A‐Nas group were significantly elevated, while the protein expression level of ACSL4 was significantly reduced compared with that in the EP group (*p *< 0.05), and similarly, compared with the EP group, the protein expression levels of GPX4 and SLC7A11 in the EP+BoNT/A‐Hip group were significantly elevated, while the ACSL4 protein expression level was significantly reduced (*p *< 0.05). However, there was no significant difference in the expression levels of GPX4, ACSL4, and SLC7A11 proteins between the EP+BoNT/A‐Nas and EP+BoNT/A‐Hip groups.

Fe^2+^, GSH, and MDA contents in the hippocampal tissues of rats in each group were detected by biochemical kits (Figure 3F–H). Compared with the control group, the Fe^2+^ and MDA contents in the EP group were significantly higher, while the GSH content was significantly lower (*p *< 0.05). Between the EP and EP+NS groups, Fe^2+^, GSH, and MDA contents did not show significant differences. In contrast, Fe^2+^ and MDA contents were significantly lower, while GSH contents were significantly higher in the EP+BoNT/A‐Nas group compared to the EP group (*p *< 0.05). Similarly, Fe^2+^ and MDA contents were significantly lower while GSH contents were significantly higher in the hippocampal tissues of rats in the EP+BoNT/A‐Hip group compared to the EP group (*p *< 0.05). However, there were no significant differences in Fe^2+^, GSH, and MDA contents between the EP+BoNT/A‐Nas and EP+BoNT/A‐Hip groups. In conclusion, BoNT/A has the ability to reduce the ferroptosis level and protect hippocampal tissues from EP‐induced injury.

### 3.5 BoNT/A Inhibits Magnesium‐Free Induction Solution‐Induced Cell Damage in Hippocampal Neurons

**FIGURE 4:**
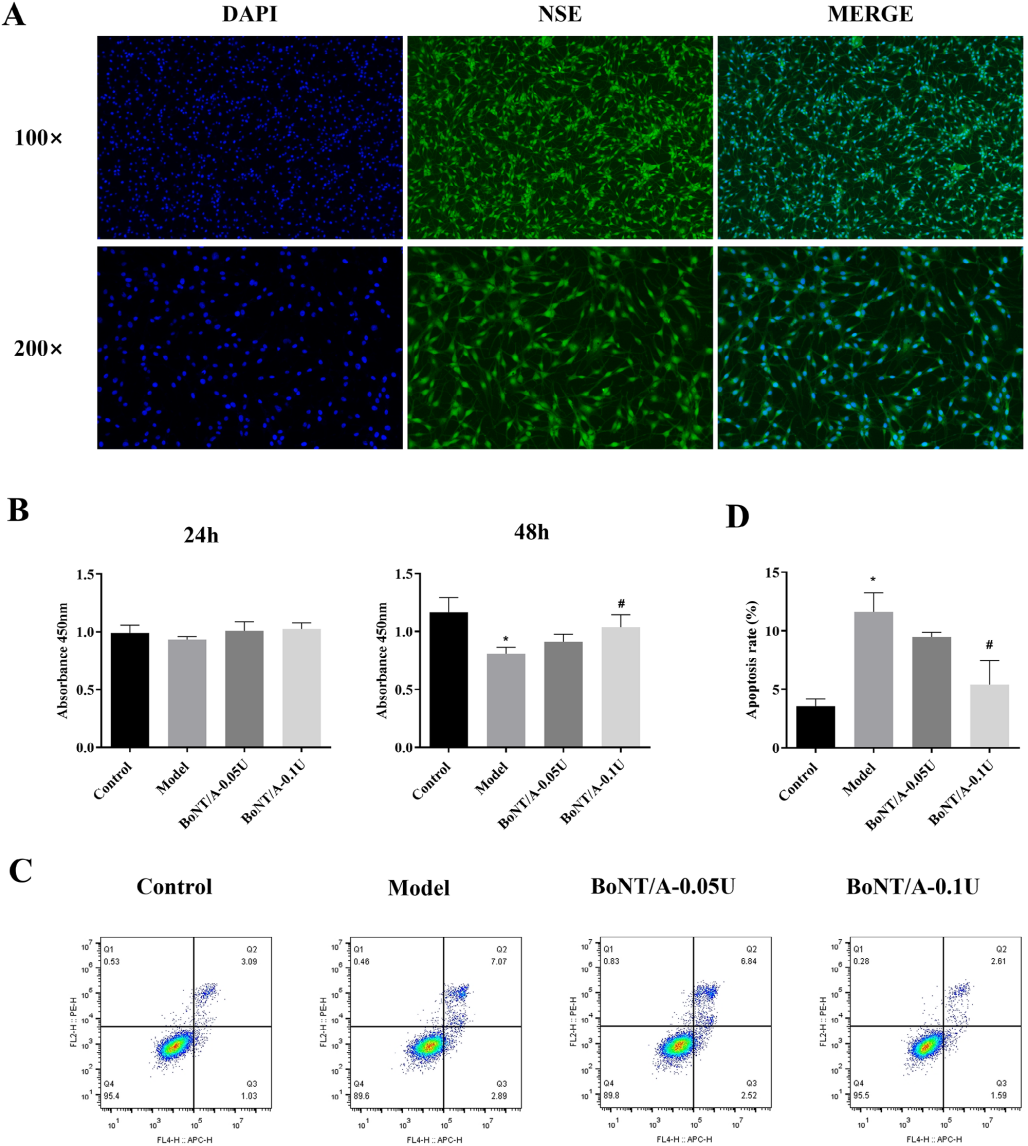
BoNT/A inhibits magnesium‐free induction solution‐induced hippocampal neuronal cell injury. (A) Identification of primary hippocampal neuronal cells. DAPI (blue) was used as a nuclear marker, and NSE (green) was used as a hippocampal neuronal cell marker. (B) CCK‐8 assay for cell proliferation activity after 24 and 48 h of culture. (C) Apoptosis rate in each group as shown by flow cytometry results. (D) Histogram of apoptosis rate results in each group. Compared with the control group, *p < 0.05; compared with the EP group, ^#^ *p *< 0.05.

After isolation of rat primary hippocampal metameric cells, immunofluorescence staining was performed using NSE antibody, which showed that almost all the detected cells were labeled with NSE (Figure 4A), indicating that the hippocampal neurons had a high degree of purity. An in vitro model of epilepsy was established by treating hippocampal neuronal cells with magnesium‐free induction solution, followed by the addition of BoNT/A for intervention. Cleaved‐SNAP25 protein expression was first detected by WB, and the results showed that Cleaved SNAP25 protein was highly expressed in the BoNT/A‐intervened groups and not in other groups (Figure S2), suggesting that BoNT/A can successfully intervene in rat hippocampal neuronal cells.

To assess the effect of BoNT/A on cell injury induced by magnesium‐free induction solution, cell proliferation activity was detected by CCK‐8 assay after 24 and 48 h of intervention (Figure 4B). The 24 h results showed that there was no significant difference in cell proliferation activity among groups. The 48 h results showed that cell proliferation activity in the model group was significantly lower than that in the control group (*p *< 0.05). Compared with the model group, cell proliferation activity was elevated in the 0.05 U BoNT/A intervention group, but the difference was not significant. Compared with the model group, cell proliferation activity was significantly elevated in the 0.1 U BoNT/A intervention group, and the difference was significant (*p *< 0.05). The above results suggest that magnesium‐free inducing solution can induce a decrease in the proliferative activity of hippocampal neuron cells, which leads to fine damage, while BoNT/A intervention can inhibit the cell damage induced by magnesium‐free inducing solution.

The apoptosis rate after 48 h of intervention was detected by flow cytometry (Figure 4C,D). The apoptosis rate in the model group was significantly higher than that in the control group, and the difference was statistically significant (*p *< 0.05). Compared with the model group, the apoptosis rate was reduced in the 0.05 U BoNT/A intervention group, but the difference was not statistically significant. Compared with the model group, the apoptosis rate was significantly reduced in the 0.1 U BoNT/A intervention group, and the difference was statistically significant (*p *< 0.05). The above results suggest that magnesium‐free inducing solution can induce a higher apoptosis rate of hippocampal neurons, while BoNT/A intervention can inhibit the apoptosis induced by magnesium‐free inducing solution.

### 3.6 BoNT/A Inhibits Magnesium‐Free Induction Solution‐Induced Oxidative Stress and Iron Death in Hippocampal Neuronal Cells

**FIGURE 5:**
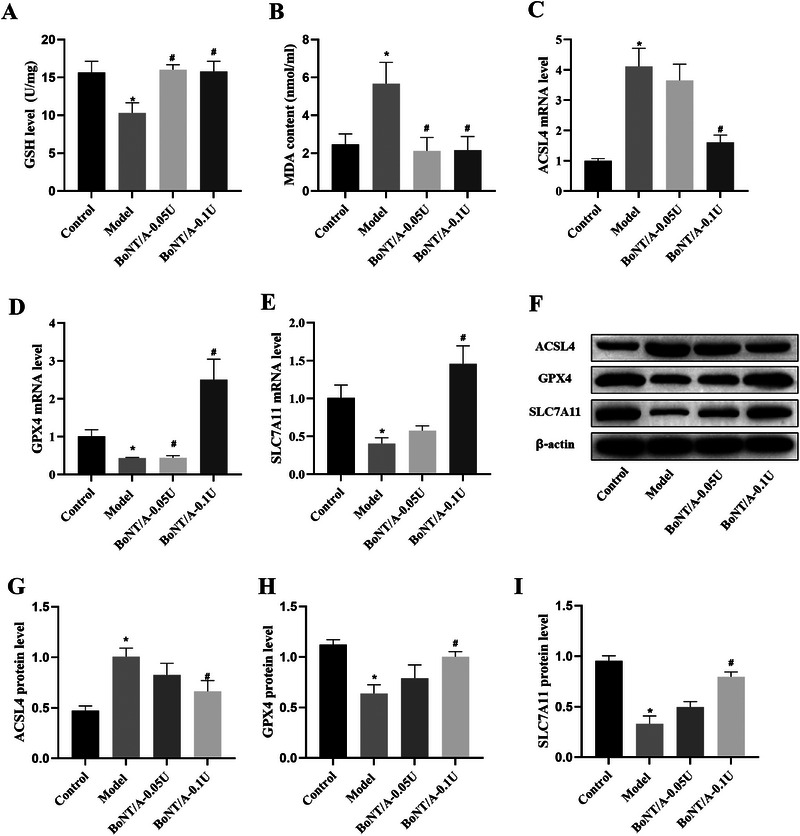
BoNT/A inhibits magnesium‐free induction solution‐induced cell damage in hippocampal neurons. (A) GSH levels in each group. (B) MDA levels in each group. (C) ACLS4 mRNA levels in each group. (D) ACLS4 mRNA levels in each group. (E) SLC7A11 mRNA levels in each group. (F) Representative WB assay strip plots. (G–I) Bar graph of ACSL4, GPX4, and SLC7A11 protein expression. Compared with the control group, *p < 0.05; compared with the EP group, ^#^ *p *< 0.05.

To assess the effect of BoNT/A on the oxidative stress induced by magnesium‐free induction solution in hippocampal neuron cells, GSH and MDA levels in the cells were detected by the kit (Figure 5A,B). The intracellular GSH level in the model group was significantly lower than that in the control group (*p *< 0.05), whereas the intracellular MDA level in the model group was significantly higher than that in the control group (*p *< 0.05). The intracellular GSH content was significantly higher in the 0.05 U BoNT/A intervention group compared to the model group, while the MDA content was significantly lower (*p *< 0.05). Similarly, intracellular GSH content was also significantly higher and MDA content was significantly lower in the 0.1U BoNT/A intervention group compared to the model group (*p *< 0.05).

To assess the effect of BoNT/A on ferroptosis induced by magnesium‐free induction solution in hippocampal neuronal cells, mRNA and protein levels of GPX4, SLC7A11, and ACSL4 were detected by qPCR and WB. qPCR results showed that GPX4 and SLC7A11 mRNA expression levels were significantly lower in the model group than that of the control group, whereas the ACSL4 mRNA expression level was significantly higher than that of the control group (*p *< 0.05, Figure 5C–E). Compared with the model group, GPX4 and SLC7A11 mRNA expression levels were significantly higher, while the ACSL4 mRNA expression level was significantly lower in the 0.05 U BoNT/A intervention group (*p *< 0.05). Similarly, compared with the model group, both GPX4 and SLC7A11 mRNA expression levels were significantly higher in the 0.1 U BoNT/A intervention group, whereas ACSL4 mRNA expression level was also significantly lower (*p *< 0.05). The results of the WB assay showed (Figure 5F–I) that the GPX4 and SLC7A11 protein expression levels were significantly lower than those in the control group, while ACSL4 protein expression levels were significantly higher than those in the control group (*p *< 0.05). Compared with the model group, the expression levels of GPX4 and SLC7A11 proteins in the cells of the 0.05 U BoNT/A intervention group were significantly higher, while the expression levels of ACSL4 proteins were significantly lower (*p *< 0.05). Similarly, the 0.1U BoNT/A intervention group also showed significantly higher levels of GPX4 and SLC7A11 protein expression and significantly lower levels of ACSL4 protein expression compared with the model group (*p *< 0.05). In conclusion, BoNT/A intervention can inhibit the ferroptosis phenomenon that was induced by magnesium‐free induction solution in neuronal cells.

## 4 Discussion

The Racine Scale (RS) is a specialized tool used to assess seizure severity and its correlates in patients with epilepsy (Luttjohann et al. 2009), and the Racine Score is particularly suitable for quantifying the behavioral performance of animals during seizures (Rook et al. 2013). Our results showed that the Racine score was significantly reduced in EP rats either by hippocampal stereotaxic injection or nasal drip, a finding suggesting that BoNT/A has the potential to suppress seizures.

The Morris water maze and the open field test are important tools for assessing cognitive behavior in epileptic rats (Bocca Nejm et al. 2021). Epileptic seizures may have effects on spatial memory and learning ability, and the Morris water maze helps to study these changes. Our results showed that after BoNT/A intervention was administered to SE rats by both hippocampal stereotaxic injection and nasal drip, the results of the Morris water maze showed a significant increase in the residence time in the target quadrant and the number of times crossing the platform. This phenomenon suggests that BoNT/A intervention may have improved the impaired spatial memory ability of EP rats. Meanwhile, it was also observed in the open field test that the rats' spontaneous activity time was significantly prolonged, which further supported the hypothesis of BoNT/A's effect on cognitive function recovery. This finding suggests that BoNT/A intervention can effectively restore cognitive dysfunction in EP rats. Meanwhile, it is also important to decipher whether BoNT/A's impact on cognitive function and hippocampal damage repair is primarily a direct neuroprotective effect or secondary to its seizure‐suppressing capabilities, which will contribute to the therapeutic strategy development of BoNT/A in the future.

Nissl staining with Golgi staining is an important technical tool for assessing cell damage in hippocampal neurons. By observing changes in the cell body, Nissl staining helps to identify neuronal damage, death, or functional changes and is often applied to detect neuronal survival and cell density. Golgi staining, on the other hand, can effectively label neuronal dendrites and synapses, making their morphologic structure clearer, which is ideal for studying neuronal protrusions and synaptic connections. Our results showed that the hippocampal neurons of EP rats were missing, and the arrangement of the neurons was disorganized, and the overall coloration was light. In addition, the total density of dendritic spines and the density of mature dendritic spines were significantly reduced, suggesting impaired neuroplasticity. After BoNT/A intervention, these abnormalities were effectively reversed, suggesting that BoNT/A intervention could alleviate the cellular damage of hippocampal neurons in EP rats. In the in vitro experiments, the treatment of magnesium‐free induction solution led to a decrease in the proliferative activity and apoptotic rate of rat hippocampal neurons, and the intervention of BoNT/A restored the proliferative activity and inhibited apoptosis of the cells. This suggests that BoNT/A intervention can inhibit EP‐induced hippocampal neuronal cell damage. At the same time, it is necessary to note that BoNT/A can also have a long‐term memory impairment effect in rats with different doses (Lackovic et al. 2009), indicating the double‐edge effect of BoNT/A on neuronal cells. These results indicate that we should do more experiments to confirm the safe concentration range of BoNT/A in rats and further validate this dose range, as well as the function and therapeutic value of BoNT/A in human samples.

Fe^2^⁺, GSH, and MDA play crucial roles in the study of cellular iron death. Fe^2^⁺ is considered to be a central factor in iron death, and its accumulation in the cell promotes lipid peroxidation, which in turn triggers iron death (Li et al. 2020; Xing et al. 2022). GSH, as an important antioxidant in iron death, mainly protects cells by inhibiting lipid peroxidation, while a decrease in its level accelerates iron death (Xu et al. 2022; Hu et al. 2022). In addition, MDA is a product in the lipid peroxidation reaction and can be used to assess the degree of intracellular lipid peroxidation. Elevated MDA levels indicate increased oxidative damage associated with iron death (Jiang et al. 2023; Wu et al. 2022). The results of our in vivo study showed that the levels of Fe^2^⁺ and MDA were significantly elevated in the hippocampal tissues of SE rats, whereas the levels of GSH were significantly reduced. After BoNT/A intervention, the levels of Fe^2^⁺ and MDA in the hippocampal tissues of EP rats were significantly decreased, while the levels of GSH were significantly increased. Similarly, in in vitro experiments, MDA was significantly elevated while GSH was significantly decreased in hippocampal neuronal cells of rats treated with no magnesium‐inducing solution. And BoNT/A intervention reversed this change. This finding suggests that BoNT/A intervention can effectively modulate the levels of Fe^2^⁺, GSH, and MDA in the hippocampal tissues of EP rats, thereby alleviating the cellular damage suffered by their hippocampal neurons.

During ferroptosis, mitochondria usually swell, and their membrane structure may be altered, leading to dilation of the inner lumen (Zhong et al. 2023). This swelling is closely related to the damage and dysfunction of the mitochondrial membrane, and in particular, it is importantly linked to the iron‐dependent lipid peroxidation reaction (Liu et al. 2023). In addition, the inner mitochondrial membrane may suffer disruption or dissociation, leading to loss of membrane integrity. Lipid peroxidation allows the mitochondrial membrane to be damaged, thus significantly affecting its function and morphology. Our results showed that EP rat hippocampal tissues had blurred nuclear membrane structure and a reduced number of organelles, while mitochondria appeared swollen and decreased in number with vacuole‐like changes. In contrast, after BoNT/A intervention, it was observed that the cell swelling was significantly reduced, the membrane structure remained intact, and the overall cell morphology was normalized.

GPX4 is a key regulatory protein for iron death and is responsible for reducing lipid peroxides, thereby preventing oxidative damage in cells (Liang et al. 2023). ACSL4 is involved in lipid metabolism and promotes the accumulation of polyunsaturated fatty acids, which increases the risk of lipid peroxidation and further contributes to the process of iron death (Xue et al. 2023). SLC7A11 is an important transporter protein for glutathione synthesis and regulates intracellular glutathione levels, and its inhibition leads to a decrease in antioxidant capacity, which promotes the development of iron death (Koppula et al. 2021). Our in vivo results showed that GPX4 and SLC7A11 protein expression levels were significantly reduced in hippocampal tissues of EP rats, whereas ACSL4 protein expression levels were significantly increased. After BoNT/A intervention, the expression levels of GPX4 and SLC7A11 proteins in the hippocampal tissues of EP rats were significantly increased, while the expression level of ACSL4 protein was significantly increased. This finding suggests that BoNT/A intervention can effectively regulate the levels of GPX4, ACSL4, and SLC7A11 in the hippocampal tissues of EP rats, thereby inhibiting iron death in EP rats. Similarly, the results of in vitro studies showed that the levels of GPX4 and SLC7A11 protein expression were significantly reduced in cells treated with magnesium‐free induction solution, whereas the level of ACSL4 protein expression was significantly increased. After BoNT/A intervention, the expression levels of GPX4 and SLC7A11 proteins in the hippocampal tissues of EP rats were significantly increased, while the expression level of ACSL4 protein was significantly increased. This suggests that BoNT/A intervention can inhibit iron death of hippocampal neuronal cells caused by magnesium‐free induction solution. However, it is not clear how BoNT/A regulates the expression levels of these iron‐associated proteins. Dose BoNT/A modulate the proteins at transcriptional or posttranscriptional levels through molecular signaling pathways? Further studies that focus on this potential direct or indirect mechanistic link would greatly enhance the understanding of how BoNT/A specifically exerts its anti‐ferroptotic effects in the context of epilepsy.

Taken together, our in vivo findings suggest that BoNT/A intervention alleviates epileptic symptoms, improves cognitive behavior, and mitigates hippocampal cell damage in EP rats by inhibiting cellular ferroptosis. In addition, the in vitro findings suggest that BoNT/A alleviates cellular damage caused by magnesium‐free induction solution by inhibiting cellular ferroptosis.

## Author Contributions

Conception and design of the research: Shuang Li and Yajun Lian. Acquisition of data: Yunqing Ma, Maiqi Du, Zhi Huang, and Yake Zheng. Analysis and interpretation of data: Shuang Li. Statistical analysis: Shuang Li, Zhi Huang, and Yake Zheng. Obtaining funding: Yajun Lian. Drafting the manuscript: Shuang Li. Revision of manuscript for important intellectual content: Yajun Lian.

## Conflicts of Interest

The authors declare no conflicts of interest.

## Supporting information




**Supplementary Figures**: brb370930‐sup‐0001‐Figures.docx

## Data Availability

The data that support the findings of this study are available from the corresponding author upon reasonable request.
