# Supplementary material for: Botulinum Toxin Type A as a Therapeutic Agent in Epilepsy: Attenuation of Neuronal Ferroptosis and Cognitive Dysfunction
Source: Brain Behav. 2025 Nov 12;15(11):e70930. doi: 10.1002/brb3.70930 (PMC12612554; doi:10.1002/brb3.70930)
Supplement: Supplementary file 1 — Supplementary Figures: brb370930‐sup‐0001‐Figures.docx [file BRB3-15-e70930-s001.docx]

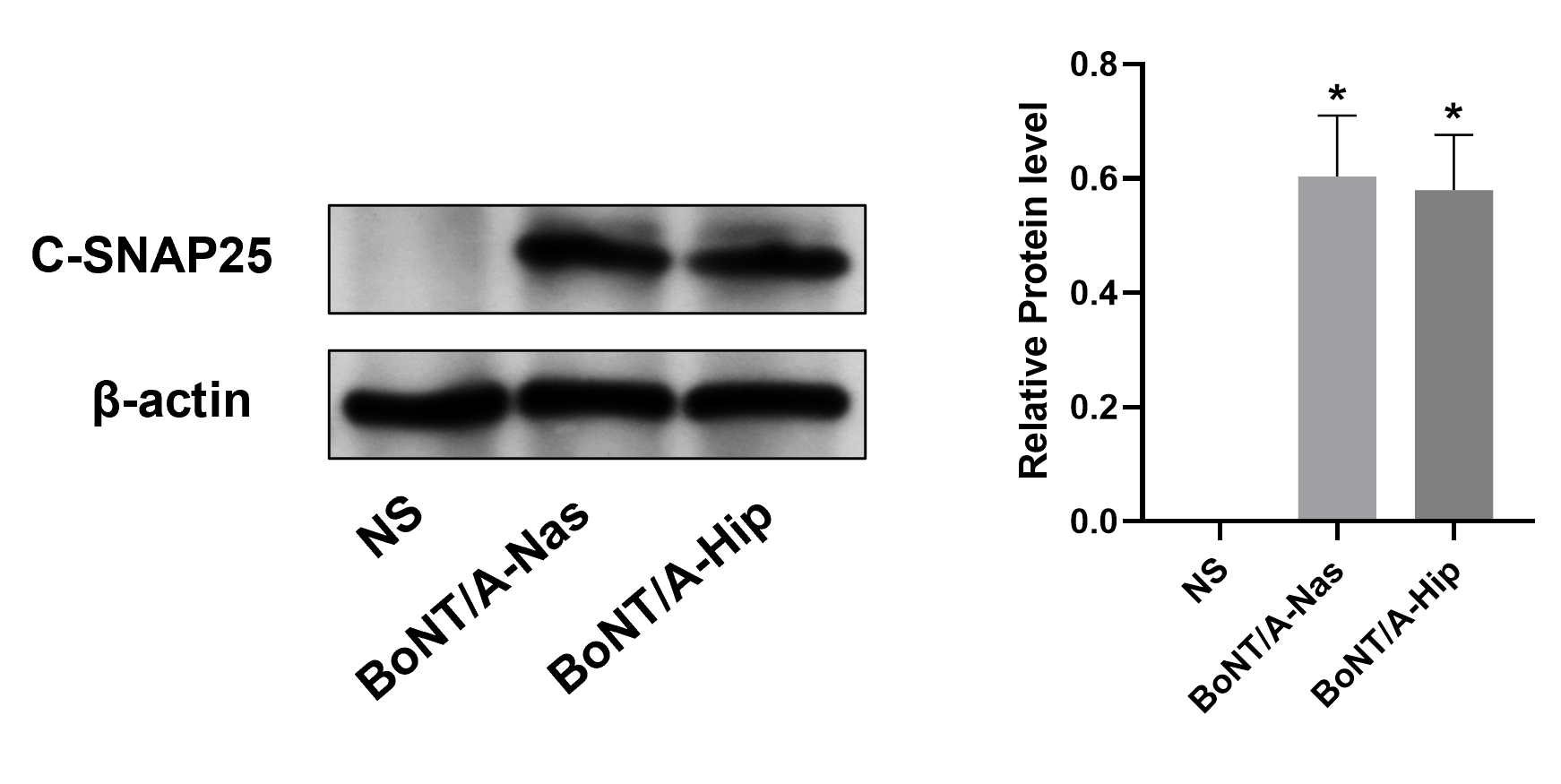


Figure S1. BoNT/A treatment cleaved SNAP25 protein in the hippocampal tissues of rats.


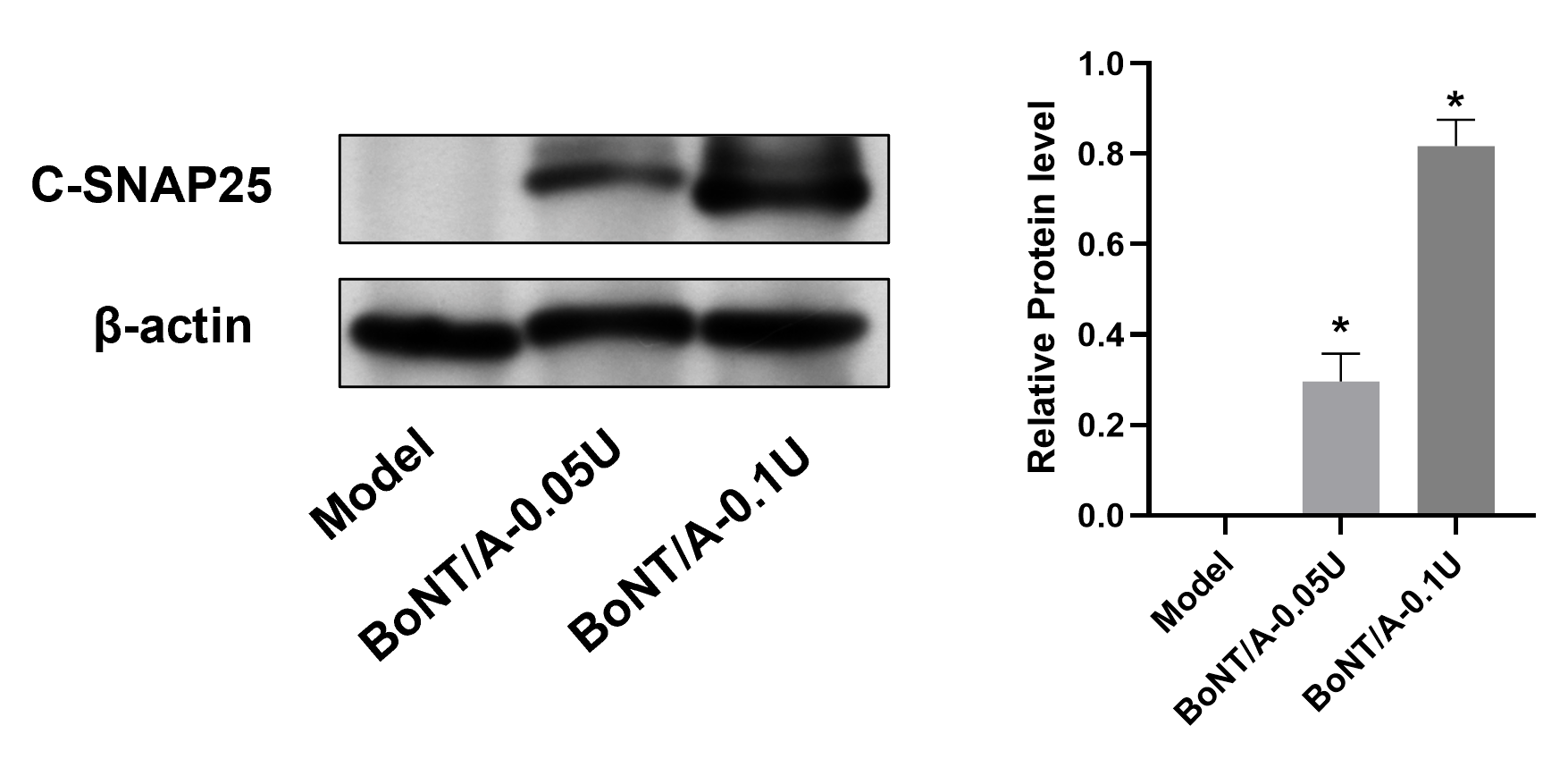


Figure S2. BoNT/A treatment cleaved SNAP25 protein in the hippocampal neuron cells of rats.
